# Association between perinatal factors and hypospadias in newborns: a retrospective case–control study of 42,244 male infants

**DOI:** 10.1186/s12884-022-04906-6

**Published:** 2022-07-20

**Authors:** Yi Wang, Lin Wang, Zeyong Yang, Fang Chen, Zhiwei Liu, Zheng Tang

**Affiliations:** 1 Department of Neonatology, International Peace Maternity and Child Health Hospital, Shanghai Jiao Tong University School of Medicine, Shanghai, China; 2 Shanghai Key Laboratory of Embryo Original Disease, Shanghai, China; 3 Shanghai Municipal Key Clinical Specialty, Shanghai, China; 4 Department of Urology, Shanghai Sixth People’s Hospital, Shanghai Jiao Tong University, Shanghai, China; 5 Department of Urology, Shanghai Children’s Hospital, Shanghai Jiao Tong University, Shanghai, China; 6 Shanghai Eastern Institute of Urologic Reconstruction, Shanghai, China; 7 Department of Anesthesiology, International Peace Maternity and Child Health Hospital, Shanghai Jiao Tong University School of Medicine, Shanghai, China

**Keywords:** Hypospadias, Pregnancy–induced hypertension hypertensive disorders of pregnancy, Hyperthyroidism, Multiple births

## Abstract

**Background:**

Hypospadias is one of the most common male congenital malformations worldwide. It is characterised by the abnormal positioning of the opening of urethra, and may lead to problems with urination and sexual function. Various factors were suggested to contribute to hypospadias pathogen. This study aimed to evaluate the relationship between perinatal factors and neonatal hypospadias based on a large sample of male newborns.

**Methods:**

This retrospective case–control study was conducted at the International Peace Maternal and Child Health Hospital, Shanghai Jiao Tong University School of Medicine. Male infants with hypospadias (*N* = 97) and without any birth defects (*N* = 42,147) who were born in January 2015 to December 2019 were enrolled in this study. A statistical analysis of perinatal factors, such as maternal age, primiparity, multiple births, hypertensive disorders of pregnancy (HDP), diabetes mellitus (DM), placenta previa, thyroid diseases, hepatitis B, obesity, meconium-stained amniotic fluid, gestational age, low birth weight (LBW), small for gestational age (SGA) and in vitro fertilization (IVF) was used to assess the risk factors for hypospadias.

**Results:**

The overall incidence of hypospadias in male infants was 0.23% (97/42,244). The univariate analysis of potential risk factors for hypospadias showed that HDP, primiparity, multiple births, hyperthyroidism, preterm delivery, LBW and SGA had a statistical association with hypospadias. After adjusting for potential confounders in a multivariate regression analysis, the odds ratios (OR) and 95% confidence intervals (CI) were calculated for the following risk factors for hypospadias: HDP (OR: 3.965, 95% CI: 2.473–6.359, *P* <  0.01), multiple births (OR: 2.607, 95% CI: 1.505–4. 514, P <  0.01) and hyperthyroidism (OR:4.792, 95% CI: 1.700–13.506, P <  0.01), which suggested these factors were significant independent risk factors for hypospadias.

**Conclusions:**

Perinatal factors, such as HDP, multiple births and hyperthyroidism may be associated with hypospadias in male infants.

## Background

Hypospadias is one of the most common male congenital malformations worldwide. It is defined by an abnormal urethral opening, and it is characterised by an abortive development of the urethral spongiosum and ventral prepuce; it may be accompanied by penile chordee, and an absence of the urethra, which may lead to problems with urination and sexual function [1]. The prevalence of hypospadias varies between countries and is estimated to be about 5–50 per 10,000 births, although there is an increasing trend in prevalence [1–4]. Corrective surgeries can be performed to improve the appearance and function of the penis, but postoperative complications may occur [5]. Hypospadias may lead to serious social problems associated with genital malformation and psychological stress.

Previous research has shown that both genetic and environmental factors contribute to the pathogenesis of hypospadias. The intent of this study was to evaluate the relationship between perinatal factors and neonatal hypospadias based on a large sample of male newborns, and to provide a perspective for the clinical prevention of hypospadias.

## Methods

### Study design and population

**Fig. 1:**
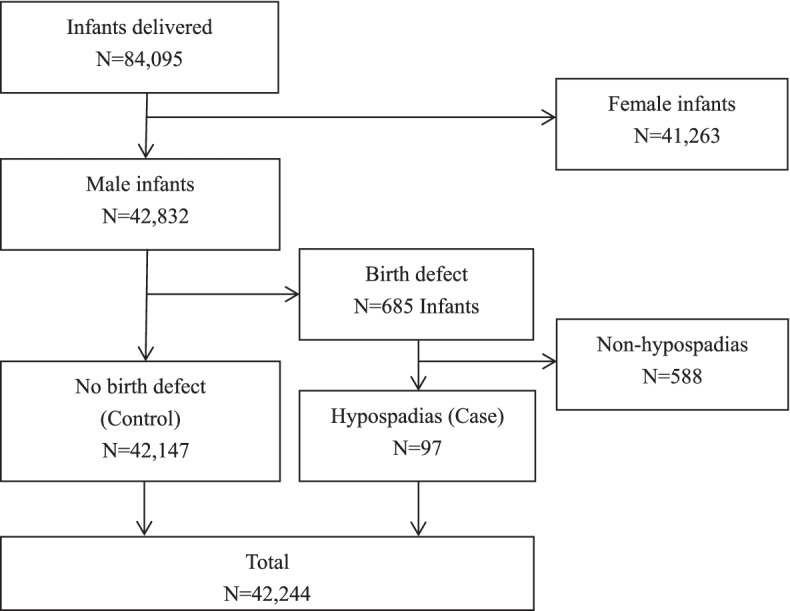
A consort flow diagram of the study. A total of 84,095 infants were born from 2015 to 2019, and 42,832 of them were male. 588 male infants with birth defects except hypospadias were excluded. Thus 42,147 male infants without birth defects (control) and 97 infants with hypospadias (case) were enrolled in this study

A retrospective case–control study was conducted on the data of pregnant women and their offspring from January 2015 to December 2019 at the International Peace Maternal and Child Health Hospital, Shanghai Jiao Tong University School of Medicine. A total of 84,095 infants were delivered over 5 years: 42,832 were male. Birth defects occurred in 685 males; 588 infants who had non–hypospadias malformations were excluded from the study. Thus, 97 infants with hypospadias formed the hypospadias case group and 42,147 male infants with no birth defects formed the control group (Fig. 1). After birth, hypospadias were diagnosed by neonatologists and urologists through clinical examinations.

### Perinatal risk factors

To determine the relationship between maternal and foetal risk factors and hypospadias, we collected information from pregnant women who underwent routine prenatal examinations and gave birth to male infants in our hospital. This information included the maternal age, primiparity, multiple births, meconium–stained amniotic fluid, gestational age (GA), newborn birth weight (BW), birth weight for GA, the use of in vitro fertilization (IVF) and diagnosis of hypertensive disorder of pregnancy (HDP), diabetes mellitus (DM), placenta previa, hyperthyroidism, hypothyroidism, hepatitis B, obesity.

The clinical information, which was completed by obstetricians and neonatologists during the prenatal examination and upon admission into the hospital, was collected from the hospital database. Diagnostic descriptions were confirmed by clinical and laboratory findings, and imaging and pathological examinations during pregnancy and after delivery according to clinical protocols and guidelines. The maternal age and primiparity were stated by the patient and confirmed by clinical records. Multiple births were diagnosed using an ultrasound scan. HDP included the following: chronic hypertension, which was diagnosed by blood pressure ≥ 140/90 mmHg before 20 weeks of gestation; gestational hypertension, which appeared after 20 weeks of gestation without proteinuria or other findings; preeclampsia, which involved gestational hypertension accompanied by proteinuria, maternal end–organ dysfunction, and/or placental dysfunction; and superimposed preeclampsia on chronic hypertension in which the pregnant women developed proteinuria or other organ dysfunction [6]. To screen for HDP, blood pressure measurements, urine tests and target organ examinations were regular performed and evaluated. A diagnosis of DM was based on routine plasma glucose tests taken during pregnancy; both pregestational and gestational DM were considered to be potential risk factors in this study [7]. Placenta previa, which is the covering of the cervical ostium by the placenta after 32 weeks of gestation, was diagnosed using transvaginal or transabdominal ultrasound [8] . Thyroid functions were evaluated by examining the serum concentrations of the thyroid hormone free thyroxine, thyroid–stimulating hormone and anti–thyroid peroxidase antibodies, which are associated with autoimmune thyroiditis. Hepatitis B virus infection was diagnosed by detecting various serological markers, such as hepatitis B surface antigen (HBsAg), hepatitis B core antigen and HBsAg antibodies. The pregnant women were considered to be obese if their body mass index (BMI = weight/height^2^) was ≥30.0 kg/m^2^ at their first prenatal examination. There were four degrees of amniotic fluid based on appearance: clear or normal amniotic fluid; I degree (i.e., light meconium and light green); II degree (i.e., intermediate meconium, yellow–green and cloudy); and III degree (i.e., heavy meconium, brown and thick). GA was calculated from the date of the last menstrual period and confirmed by early ultrasound. A preterm delivery was a birth that took place before 37 weeks (i.e., GA < 37 weeks). The infants’ BW were measured after umbilical cord clamping; a low birth weight (LBW) was defined as < 2500 g. Additionally, infants were classified according to their BW for GA: small for GA (SGA, BW < 10th percentile); appropriate for GA (AGA, BW between the 10th and 90th percentile); and large for GA (LGA, BW > 90th percentile). Information about IVF techniques was collected from the clinical history of the mothers.

Every procedure was carried out in accordance with the relevant guidelines. Ethics approval and use of clinical data for research purposes was granted by the Ethics Committees of the International Peace Maternity and Child Health Hospital. This clinical study was registered at www.chictr.org.cn(ChiCTR2000032771).

### Statistical analysis

To evaluate the effects of the perinatal factors on hypospadias, a single factor t–test and chi–square test were used for the continuous variables and categorical variables, respectively. Directed acyclic graphs (DAGs) were depicted using DAGitty v.3.0 software to determine the minimal sufficient adjustment sets as confounding factors [9]. Nodes represented variables and arrows denoted the assumed existence and directions of causal relationships. Confounders were common causes of both the exposure and the outcome, and we used these confounders to construct a multivariate logistic regression analysis to assess the independent contribution of the variables to the risk of hypospadias. The results were presented as odds ratios (OR) and 95% confidence intervals (95% CIs). A significance criterion of *P* <  0.05 was set for all the tests. IBM SPSS Statistics Version 25.0 software was used to sort and analyse the relevant data.

## Results

From a total of 42,244 male infants, 97 were diagnosed with hypospadias; thus, the incidence of hypospadias was 23 per 10,000 male infants (97/42,244). Six infants with hypospadias had complications from other nonfatal congenital defects: three infants had congenital heart defects, specifically a ventricular septal defect (VSD); one infant had pulmonary sequestration; one had polydactylism; and one had both VSD and a cleft hand.

In this study, HDP included chronic hypertension, which developed before 20 weeks gestation, and gestational hypertension, which developed after 20 weeks gestation. The collected data showed that no case of hypospadias was found in the 119 women with chronic hypertension. However, the data for women with chronic hypertension and gestational hypertension was not adequate for analysis and, thus, HDP was not separated into the different types.

The single factor analysis showed that there was a significantly higher proportion of women with HDP in the hypospadias case group than in control group: 25.8 and 6.8% respectively (Table 1). In the 2904 women with HDP, the incidence of hypospadias in the offspring was 0.86% (25/2904), which was about 3.7 times higher than the overall incidence of hypospadias in male infants. There was also a statistically significant higher proportion (*P* <  0.01) of the following factors in the hypospadias case group than in the control group: multi births (17.5% versus 5.5%), hyperthyroidism (4.1% versus 0.6%), preterm delivery (30.9% versus 9.0%), LBW infant (34.0% versus 5.1%), and SGA infants (27.8% versus 2.0%). The effects that maternal age, primiparity, placenta previa, DM, hypothyroidism, hepatitis B, obesity, stained amniotic fluid and IVF had on hypospadias were not significant (*P* > 0.05).

**Table 1:** Single factor analysis for Hypospadias

	Hypospadias N(%)	Non-hypospadias N(%)	*p*
Total	97(100%)	42,147 (100%)	
Average age (year)	31.9(23 ~ 42)	31.0(17 ~ 49)	0.160
Hypertensive disorder of pregnancy			< 0.01
Yes	25(25.8%)	2879(6.8%)	
No	72(74.2%)	39,268(93.2%)	
Primiparity			0.100
Yes	57(58.8%)	28,088(66.6%)	
No	40(41.2%)	14,059(33.4%)	
Placenta previa			0.561
Yes	2(2.1%)	579(1.4%)	
No	95(97.9%)	41,568(98.6%)	
Multiple births			< 0.01
Yes	17(17.5%)	2307(5.5%)	
No	80(82.5%)	39,840(94.5%)	
Diabetes Mellitus			0.122
Yes	19(19.6%)	5948(14.1%)	
No	78(80.4%)	36,199(85.9%)	
Hyperthyroidism			< 0.01
Yes	4(4.1%)	243(0.6%)	
No	93(95.9%)	41,904(99.4%)	
Hypothyroidism			0.683
Yes	4(4.1%)	1422(3.4%)	
No	93(95.9%)	40,725(96.6%)	
Hepatitis B			0.494
Yes	4(4.1%)	1242(2.9%)	
No	93(95.9%)	40,905(97.1%)	
Obesity			0.300
Yes	6(6.2%)	1727(4.1%)	
No	91(93.8%)	40,420(95.9%)	
Amniotic fluid			0.279
clear	84(86.6%)	34,224(81.2%)	
I ~ II stained	7(7.2%)	3196(7.6%)	
III stained	6(6.2%)	4728(11.2%)	
Preterm delivery			< 0.01
Yes	30(30.9%)	3799(9.0%)	
No	67(69.1%)	38,348(91.0%)	
Low birth weight infant			< 0.01
Yes	33(34.0%)	2153(5.1%)	
No	64(66.0%)	39,994(94.9%)	
IVF			0.694
Yes	8(8.2%)	3040(7.2%)	
No	89(91.8%)	39,107(92.8%)	
Birth weight for gestational age			< 0.01
SGA	27(27.8%)	850(2.0%)	
AGA	60(61.9%)	30,740(72.9%)	
LGA	10(10.3%)	10,557(25.1%)	

Low birth weight infants: Birth weight < 2500 g; *IVF* In Vitro Fertilization, *SGA* Small for Gestational Age, *AGA* Appropriate for Gestational Age, *LGA* Large for Gestational Age

**Fig. 2:**
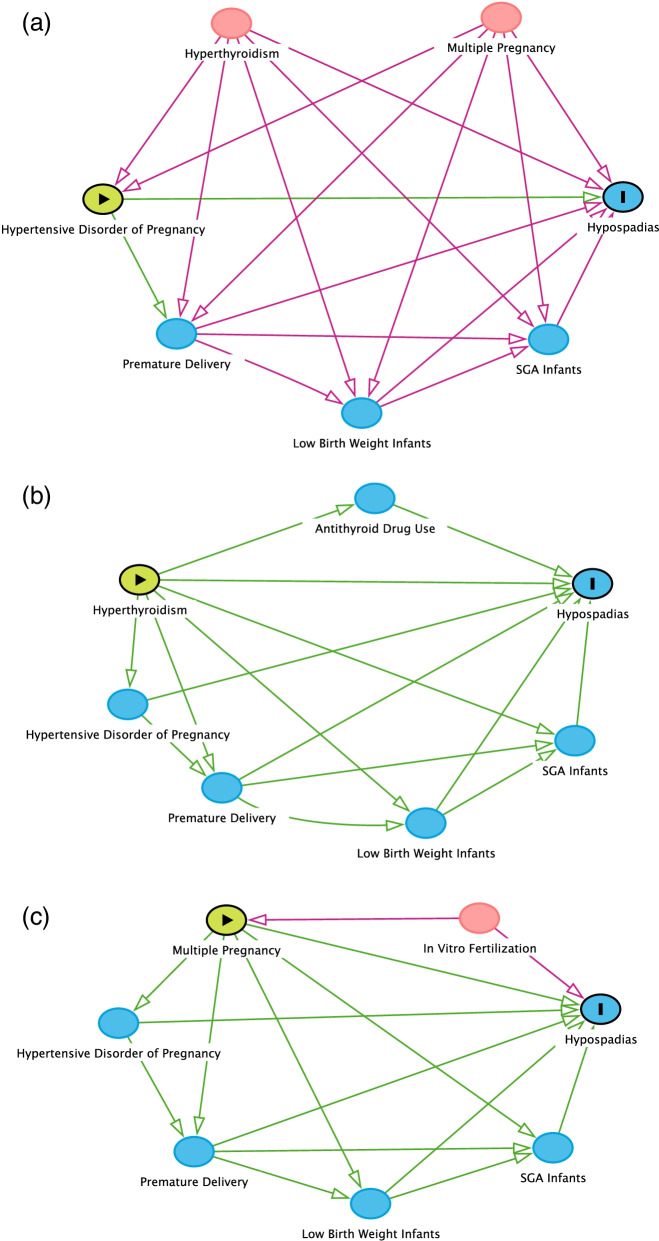
Directed Acyclic Graphs (DAGs) for identifying confounding variables. Nodes represented variables and arrows denoted the assumed existence and directions of causal relationships. **a** Hypertensive disorder of pregnancy (HDP), (**b**) hyperthyroidism and (**c**) multiple births were assumed to be exposures of the outcome, hypospadias

DAGs were depicted to determine the confounding variables for hypospadias (Fig. 2). HDP, hyperthyroidism and multiple births was assumed to be the exposures of the outcome, hypospadias. Preterm delivery, LBW infants， SGA infants and antithyroid drug use were considered to be mediators that fell on the causal paths between the exposures to outcome, so they were not confounders and they were eliminated from the multivariate analysis. Therefore, a multivariate logistic regression analysis was performed on hypospadias and the following risk factors: HDP, multiple births and hyperthyroidism (Table 2). The results showed that there were significant relationships between hypospadias HDP (OR:3.965, 95% CI: 2.473–6.359, *P* <  0.001), multiple births (OR: 2.607, 95%CI: 1.505–4.514, *P* = 0.001) and hyperthyroidism (OR: 4.792, 95% CI: 1.700–13.506, *P* = 0.003).

**Table 2:** Multivariate logistic regression analysis for hypospadias

Influence factor	Crude analysis		Adjusted analysis	
	OR (95% CI)	*p*	OR (95% CI)	*p*
HDP	4.736 (3.000–7.476)	0.000*	3.965 (2.473–6.359)	0.000*
Multiple births	3.670 (2.171–6.204)	0.000*	2.607 (1.505–4. 514)	0.001*
Hyperthyroidism	7.417 (2.705–20.339)	0.000*	4.792 (1.700–13.506)	0.003*

*OR* Odds Ratios, *CI* Confidence Intervals, *HDP* Hypertensive Disorder of Pregnancy. * *p* value < 0.01

## Discussion

Hypospadias is the most common external genital malformation in males, it mostly manifests as simultaneous defects of the urethra and corpus spongiosum [1, 10]. Males with hypospadias are usually unable to urinate while standing; the sexual function in adults may also be affected, which not only impacts fertility, but can also trigger anxiety and low self–esteem, as well as cause psychological problems for patients and their families. The current lack of understanding of the mechanisms and risk factors associated with hypospadias has complicated the prevention and treatment of the condition.

Studies have reported that the occurrence of hypospadias is related to genetic predisposition, HDP, abnormal placental cord insertion, placental dysfunction, hepatitis B antigen carrier, exposure to various medical and chemical substances, maternal age, preterm delivery, SGA and birth length [7, 10–18]. In this hospital–based retrospective case–control study of 42,244 male infants, our results indicated that there was a significant relationship between hypospadias and HDP, multiple births and hyperthyroidism. Our analysis did not support a relationship between hypospadias and maternal age and hepatitis B virus carrier; we also found that preterm delivery, LBW and SGA was not independent risk factors for hypospadias.

The result of this study showed that there was a significant increase in the incidence of hypospadias in male newborns from mothers with HDP; the incidence was 0.86%, which was about 3.7 times higher than the overall incidence of hypospadias. Moreover, after multivariate correction, the results of the analysis still showed that the risk of hypospadias caused by HDP was more than 3 times the overall incidence, which was statistically significant. HDP included chronic hypertension, gestational hypertension, preeclampsia and superimposed preeclampsia on chronic hypertension. Previous studies showed conflicting results about the relationship between different types of HDP and hypospadias [13, 19]. The timing of the onset of high blood pressure during a pregnancy may cause different outcomes for the foetus. In our study design, we categorised HDP according to the onset time of before or after 20 weeks of gestation. However, the data for mothers with chronic hypertension was not adequate for statistical analysis; furthermore, no mothers with chronic hypertension had infants with hypospadias.

The key window for the development of male reproductive organs occurs from 8 to 14 weeks of gestation [20]. One study showed evidence that the morphogenesis of the male reproductive organs depends on a series of hormonal stimulations, and that placental–derived hormones play key roles in the development of reproductive organs [20]. Initially, human chorionic gonadotropin is secreted by the placenta and stimulates the differentiation of testicular stromal cells in the embryo. By the 8th week of gestation, the testicular stromal cells begin secreting of testosterone, which is essential for the normal development of the penis and testes. This finding suggests that placental dysfunction in early pregnancy may be a possible factor that is involved in the pathogenesis of hypospadias. Another study found that placental dysfunction was common in patients with HDP, which indicated that HDP was relevant to the pathogenesis of hypospadias [21]. In many pregnant women, HDP, and especially preeclampsia, can have serous effects on early placental function. Hypertension caused by the insufficient invasion of the spiral arteries or by luminal contraction and stenosis can lead to inadequate placental blood flow perfusion, which alters placental function and affects foetal growth and development [11, 19, 21, 22]. Although gestational hypertension is usually diagnosed after 20 weeks of gestation, which is later than the key window for the development of hypospadias, its aetiology may be initiated before 20 weeks of gestation or it may be present from the beginning of gestation. This means that gestational hypertension may have an impact on the foetal development before the condition has even been diagnosed. Certainly, there is a question as to whether hypospadias is secondary to HDP or they are outcomes of a common pathogen. Further genetic or pathological studies are expected to reveal the potential pathogenesis of hypospadias.

There were significantly higher numbers of LBW, SGA and preterm infants in hypospadias case group than in the control group, which suggested that these factors might increase the risk of hypospadias. However, these factors are strongly associated with gestational complications that can lead to birth defects. For example, HDP and hyperthyroidism are common causes of severe crises in pregnancies and can affect the timing of an obstetrician’s decision to deliver the baby earlier. HDP and multiple births may contribute to chronic intrauterine distress, which can affect foetus growth and development. Therefore, preterm delivery and LBW infants are common in pregnancies with these gestational complications. These findings indicated that LBW, SGA and preterm infants were not independent risk factors for congenital malformations, but rather common outcomes of the other conditions. One study reported that birth length was significantly related to the occurrence of hypospadias [17]. While the BWs of infants differ greatly in the late stages of pregnancy, the birth length grows constantly and may reflect placental function in the early stages of pregnancy. In our study, birth length was not collected for factor analysis because the measurements depended on keeping the infant’s knees stretched out and completely straight. Thus, the birth length data might vary between different operators. A standard measurement for birth length would increase accuracy for further study to estimate foetal growth.

In our study, mothers with hyperthyroidisms during pregnancy had a significant increased risk of hypospadias in their newborns. However, the relationships between hyperthyroidism and related medical treatments and birth defects are still controversial. There were increasing evidences indicated that the drugs used to treat hyperthyroidism in early pregnancies are teratogenic. Methimazole and carbimazole exposures were believed to be associated with severe birth defects and, thus, propylthiouracil was recommended for hyperthyroidism treatment in the first trimester of pregnancy [14, 15, 23]. Further studies discovered elevated risks of birth defects in mothers who received propylthiouracil treatment; however, the risk was similar to that of untreated hyperthyroidism [24]. One study found that hyperthyroidism were significantly associated with an increased risk of foetal growth restriction, as well as a higher rate of gestational hypertension, which was also associated with hypospadias [25]. Our results showed that both hyperthyroidism and HDP were statistically significant independent risk factors for hypospadias. Future studies that further classify thyroid diseases and their treatments may provide further information about their certain relationships with hypospadias.

Previous studies have reported that multiple births are associated with an elevated risk of hypospadias; the OR ranged from 2.58 to 3.47 [18, 26]. Multiple births were considered to be associated with placental insufficiency and growth restriction, which play important roles in hypospadias pathogenesis [18]. In addition, multiple births have been increasing these years on account of assisted reproductive technology (ART) development. One study showed modest associations between ART and prevalence of birth defects including hypospadias, and multiple births were suggested to be mediator variables to explain the relative relationship between ART and birth defects [27]. Our results were consistent with previous studies on the association between multiple births and hypospadias. However, IVF, as an important technology of ART, did not show increasing risk of hypospadias in the infants, and this result was consistent with the other sturdies [26, 28, 29].

The California Birth Defects Monitoring Program and the Emilia–Romagna Registry (IMER) Program found that the risk of hypospadias was significantly increased in the offspring of older mothers [16, 28]. However, we did not reach a similar conclusion. Our results showed that there was no significant difference in maternal age between the hypospadias case group and the control group.

The limitation of our study was that it was a retrospective research from a single center. The data were collected from the hospital information system, which was not well–designed for specific researches. Only well collected factors were considered in this study. Therefore, certain data, such as information on the severity of the hypospadias, the medication treatment given for HDP and thyroid disease, whether the mother smoked or drink alcohol and the assessments of placenta function in the early stage of pregnancies, were not available. Further researches may require more refined classifications and a more comprehensive design to provide deep insight into the complex aetiology of hypospadias and more centers are expected to join in co–operation.

## Conclusion

Our findings suggested that some perinatal factors of the mother, such as, HDP, multiple births and hyperthyroidism, were independent risk factors for hypospadias in newborns. Future well–designed researches that involves co–operation between paediatricians and urologists would be expected to reveal more details about the relationship between perinatal factors and hypospadias.

## Data Availability

The datasets used or analysed during the current study are available from the corresponding author on reasonable request.

## Abbreviations

AGA: Appropriate for gestational age
ART: Assisted reproductive technology
BMI: Body mass index
CI: Confidence intervals
DAG: Directed acyclic graph
DM: Diabetes mellitus
HBsAg: Hepatitis B surface antigen
HDP: Hypertensive disorder of pregnancy
IVF: In vitro fertilization
LBW: Low birth weight
LGA: Large for gestational age
OR: Odds ratios
SGA: Small for gestational age
